# Discrepancy between pregnancy dating methods affects obstetric and neonatal outcomes: a population-based register cohort study

**DOI:** 10.1038/s41598-018-24894-y

**Published:** 2018-05-02

**Authors:** Merit Kullinger, Michaela Granfors, Helle Kieler, Alkistis Skalkidou

**Affiliations:** 1 Department of Women’s and Children’s Health, Uppsala University, Uppsala, Sweden; 2 Region Västmanland – Uppsala University, Center for Clinical Research, Hospital of Västmanland, Västerås, Sweden; 3 Department of Medicine, Clinical Epidemiology Unit, Karolinska Institutet, Stockholm, Sweden; 4 Department of Clinical Science, Karolinska Institutet, Danderyd Hospital, Stockholm, Sweden; 5 Centre for Pharmacoepidemiology (CPE), Department of Medicine, Solna, Karolinska Institutet, Stockholm, Sweden

## Abstract

To assess associations between discrepancy of pregnancy dating methods and adverse pregnancy, delivery, and neonatal outcomes, odds ratios (ORs) with 95% confidence intervals (CIs) were calculated for discrepancy categories among all singleton births from the Medical Birth Register (1995–2010) with estimated date of delivery (EDD) by last menstrual period (LMP) minus EDD by ultrasound (US) −20 to +20 days. Negative/positive discrepancy was a fetus smaller/larger than expected when dated by US (EDD postponed/changed to an earlier date). Large discrepancy was <10^th^ or >90^th^ percentile. Reference was median discrepancy ±2 days. Odds for diabetes and preeclampsia were higher in pregnancies with negative discrepancy, and for most delivery outcomes in case of large positive discrepancy (+9 to +20 days): shoulder dystocia [OR 1.16 (95% CI 1.01–1.33)] and sphincter injuries [OR 1.13 (95% CI 1.09–1.17)]. Odds for adverse neonatal outcomes were higher in large negative discrepancy (−4 to −20 days): low Apgar score [OR 1.18 (95% CI 1.09–1.27)], asphyxia [OR 1.18 (95% CI 1.11–1.25)], fetal death [OR 1.47 (95% CI 1.32–1.64)], and neonatal death [OR 2.19 (95% CI 1.91–2.50)]. In conclusion, especially, large negative discrepancy was associated with increased risks of adverse perinatal outcomes.

## Introduction

Initially, the estimated date of delivery (EDD) is generally calculated based on the first day of the last menstrual period (LMP) and may later be modified when an ultrasound (US) scan is performed. According to the International Society of Ultrasound in Obstetrics and Gynecology, clinical decisions should preferably be based on the EDD by US^1^, and based on first trimester ultrasound, if performed.

The most frequently used formula for pregnancy dating in Sweden today is based on fetal biparietal measurements during the second trimester US scan, and this formula can be used to predict the day of delivery with a standard deviation (SD) of 8 days^2–4^. A minority of clinics perform first trimester pregnancy dating, with increasing practice during the last decade^2,5^. Before 2010, the combined information from measurement of the biparietal diameter and femur length were generally used^5^. The random error for most US-based formulae is <1 week, but the natural variation in growth implies that the EDD by US is less precisely estimated in some pregnancies, which may affect how they are managed^2,3,6–8^.

Although the US-based method is superior to the LMP-based method in most pregnancies, some maternal and fetal characteristics, such as the sex of the fetus, may influence the precision of the US-based estimate, and this lack of precision may be associated with adverse perinatal outcomes^9–13^. The discrepancy between dating methods and its association with pregnancy-related outcomes has been investigated in a few studies, but these have included a small study size or a limited number of perinatal outcomes^9,10,14^.

The aim of this large population-based Swedish register study was to assess whether the discrepancy between LMP-based and US-based EDD is associated with a series of adverse pregnancy, delivery, and neonatal outcomes.

## Methods

This register-based cohort study included all singleton births, live or stillborn, in Sweden, from 1995 to 2010, with valid documentation of the EDD based on both LMP and US, and a discrepancy between estimates of 20 days or less. During the study period, US scanning was offered to all pregnant women and was accepted by >95%, and US was the recommended method for pregnancy dating^15^. According to a 1996 study of the 59 clinics in Sweden that provided obstetric and antenatal care, pregnancy dating was based on a routine US examination performed between gestational weeks 16–20 in 52 clinics, and on a US examination performed at 10–15 weeks in three clinics^5^. There was no available information on an individual basis concerning the day when the pregnancy dating by US was performed.

### Data sources

All data were retrieved from the national Medical Birth Register and the Swedish Patient Register, in which information is prospectively recorded and of good quality^15–17^. All births with a live-born infant, irrespective of gestational age (GA), were recorded in the Medical Birth Register during the entire study period. Stillbirths were recorded if occurring after 27 gestational weeks + 6 days until a change in legislation on July 1, 2008, and after 21 gestational weeks + 6 days after this date^18^. Diagnoses were recorded by physicians or midwives according to the International Classification of Diseases (ICD). During the study period, the ninth version (ICD-9) was used until 1997, and the 10^th^ version (ICD-10) was used after 1997 (Supplementary Table 1).

**Figure 1:**
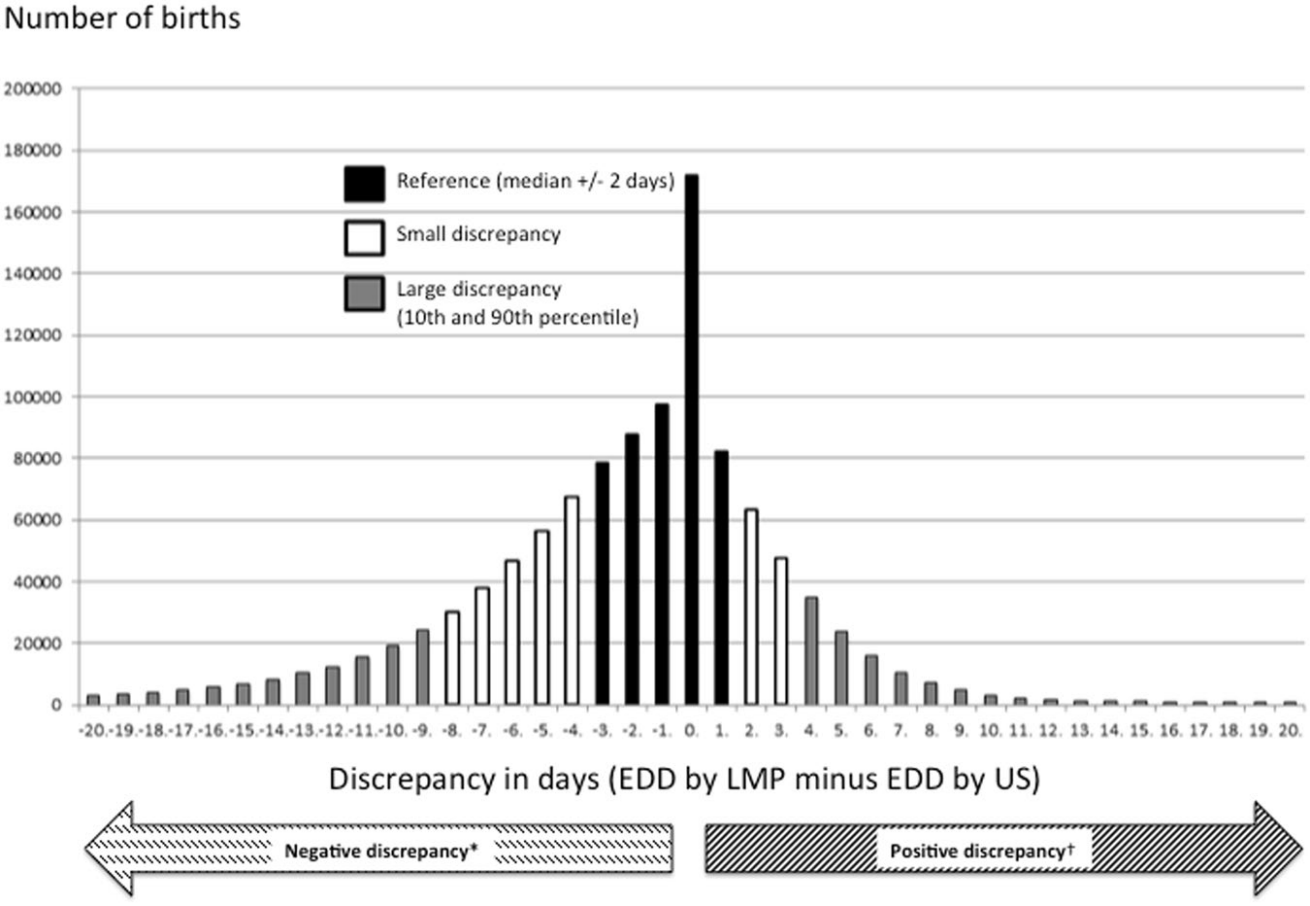
Number of births (n = 1 100 049) and discrepancy in days defined as the estimated date of delivery (EDD) by last menstrual period LMP minus EDD by ultrasound (US).

A discrepancy in days was defined as the EDD by LMP minus the EDD by US. *Negative discrepancy* was defined as the EDD by LMP earlier than the EDD by US; that is, a more advanced GA estimated by LMP than by US. The fetus was therefore *smaller than expected* when dated by US, and the EDD was postponed. *Positive discrepancy* was defined as the EDD by LMP later than the EDD by US, which corresponded to a less advanced GA estimated by LMP than by US. The fetus was therefore *larger than expected* when dated by US, and the EDD was changed to an earlier date. A large discrepancy was defined as below the 10^th^ percentile (large negative discrepancy) and above the 90^th^ percentile (large positive discrepancy) in the discrepancy distribution. The reference category was defined as a discrepancy within 2 days of the median. The remaining pregnancies were defined as a small negative or small positive discrepancy (Fig. 1).

*Pregnancy outcomes* were selected as those possibly related to fetal size, and included preeclampsia (6424, 6425, O14) or diabetes mellitus (6480, O24)^19,20^ (Supplementary Table 1).

*Delivery outcomes* were included if adverse outcomes were expected to be more frequent among large infants at birth because a larger fetal size may be apparent at the time of the dating scan: prolonged second stage of labor (6622, O63.1), delivery by forceps or vacuum extractor (6695, O81), delivery by cesarean section (6697, O82), shoulder dystocia (6604, O66.0), postpartum hemorrhage (666, O72), and perineal laceration: third or fourth degree (6642, 6643, O70.2, O70.3)^21–23^ (Supplementary Table 1).

Neonatal outcomes were included as outcomes related to growth deviations that may be present at the time of the dating scan. These outcomes included Apgar score <7 at 5 minutes, birth asphyxia (7685, P21.0), intrauterine fetal death, and neonatal death^3^ (Supplementary Table 1). To check for any association with the discrepancy between dating methods to infant size at birth, we included small for gestational age (SGA) and large for gestational age (LGA). SGA and LGA were defined as more than two SDs from the expected mean birth weight for fetal sex according to ultrasound-based GA, which reflects the clinical practice in Sweden, and is equivalent to below and above the second to third percentile^24^.

Multiple logistic regression analyses were used to calculate crude and adjusted odds ratios (ORs) and their 95% confidence intervals (95% CIs) in a crude model and in four adjusted models (models 1–4). In model 1, we adjusted for body mass index (BMI, weight (kg)/height (m^2^) <18.5 kg/m^2^ or >30 kg/m^2^, maternal age <20 years or >30 years, smoking or snuff use, living without a partner, and not being employed. In model 2, fetal sex was added to the first model with female as the reference category. In model 3, a diagnosis of diabetes mellitus or preeclampsia recorded during the current pregnancy was added as a covariate to those included in model 2. In model 4, SGA or LGA at birth was added to the covariates included in model 3. A sensitivity analysis was performed in the crude model and in models 1–3 after exclusion of SGA and LGA births. Numbers of events, event rates, risk differences (the difference in event rate between large discrepancy and reference categories), and numbers needed to treat (NNT = 1/risk difference) were calculated for the two large discrepancy categories in relation to the reference category. NNT should in this context be interpreted as number needed to follow up more closely to possibly detect the specific adverse outcome. When NNT <1, the adverse outcome is expected to be less prevalent than in the reference category, and these numbers were omitted.

Statistical analyses were performed using R statistical software version 3.1.3 (R Core Team (2015)).

The Regional Ethical Review Board in Uppsala, Sweden, approved the study protocol (reference number 2012/412, December 19, 2012).

### Ethical statement

The study was carried out according to STROBE guidelines for observational studies. The Regional Ethical Review Board in Uppsala, Sweden, approved the study protocol (reference number 2012/412, December 19, 2012). Informed consent was not possible, as it is normally not allowed in national register studies, because contacting individuals would interfere with personal integrity and the ethical board solely granted access to de-identified data.

## Results

All births in the study population (n = 1 201 679) were categorized into those with a large negative discrepancy (n = 119 275), small negative discrepancy (n = 238 929), reference category (n = 517 657), small positive discrepancy (n = 110 952), or large positive discrepancy (n = 113 236). The mean maternal age was 30 years (SD = 5 years), mean height was 166 cm (SD = 6 cm), and mean BMI was 24 kg/m^2^ (SD = 4 kg/m^2^). The median GA at birth by ultrasound was 280 days (range 154–321 days).

### Pregnancy outcomes

The odds for preeclampsia or diabetes mellitus were higher for cases of *negative discrepancy* (Table 1). The odds for diabetes were lower for cases of *positive discrepancy* (Table 1). The results remained significant after adjustments in all four models (Supplementary Table 2) and after exclusion of SGA and LGA births (data not shown).

**Table 1:** Adverse obstetric and neonatal outcomes according to the discrepancy between pregnancy dating methods (n = 1 100 049).

	Prevalence (n/100 000 births)	Large negative discrepancy n = 119 275		Small negative discrepancy n = 238 929		Small positive discrepancy n = 110 952		Large positive discrepancy n = 113 236	
		OR	(95% CI)	OR	(95% CI)	OR	(95% CI)	OR	(95% CI)
**Pregnancy or delivery outcomes**									
Preeclampsia	3377	1.23	(1.19–1.27)	1.13	(1.10–1.16)	0.96	(0.93–1.00)	0.97	(0.94–1.01)
Diabetes mellitus in pregnancy	1459	1.46	(1.39–1.53)	1.20	(1.16–1.25)	0.87	(0.82–0.93)	0.86	(0.81–0.91)
Prolonged second stage of labor	720	0.90	(0.84–0.98)	0.88	(0.83–0.94)	1.10	(1.02–1.18)	1.11	(1.03–1.19)
Delivery by forceps or vacuum extractor*	6564	0.88	(0.85–0.90)	0.90	(0.88–0.92)	1.10	(1.07–1.13)	1.11	(1.09–1.14)
Delivery by cesarean section	13289	0.92	(0.90–0.94)	0.93	(0.92–0.95)	1.02	(1.00–1.03)	1.00	(0.98–1.02)
Delivery by emergency cesarean section**	6778	0.95	(0.93–0.97)	0.95	(0.94–0.97)	1.03	(1.00–1.06)	1.02	(1.00–1.05)
Shoulder dystocia*	202	1.12	(0.97–1.28)	0.95	(0.85–1.07)	1.02	(0.88–1.18)	1.16	(1.01–1.33)
Postpartum hemorrhage	5661	0.92	(0.90–0.95)	0.93	(0.91–0.95)	1.06	(1.04–1.09)	1.13	(1.10–1.16)
Perineal laceration: third or fourth degree*	3100	0.85	(0.82–0.88)	0.88	(0.85–0.90)	1.12	(1.08–1.16)	1.13	(1.09–1.17)
**Neonatal outcomes**									
Apgar score < 7 at 5 minutes	557	1.18	(1.09–1.27)	1.03	(0.97–1.10)	0.91	(0.83–1.00)	1.00	(0.91–1.10)
Birth asphyxia	987	1.18	(1.11–1.25)	1.03	(0.98–1.08)	1.02	(0.96–1.09)	1.07	(1.00–1.14)
Intrauterine fetal death	274	1.47	(1.32–1.64)	1.24	(1.14–1.36)	0.87	(0.75–0.99)	0.89	(0.77–1.01)
Neonatal death (<28 days)	141	2.19	(1.91–2.50)	1.26	(1.10–1.43)	0.89	(0.73–1.08)	1.00	(0.82–1.20)
Small for gestational age (<2 SDs)	2169	1.54	(1.48–1.60)	1.31	(1.26–1.35)	0.81	(0.77–0.86)	0.82	(0.78–0.87)
Large for gestational age (>2 SDs)	3617	1.10	(1.06–1.14)	1.04	(1.01–1.07)	1.02	(0.99–1.06)	1.02	(0.99–1.06)

Logistic regression-derived crude ORs and 95% CIs for adverse obstetric and neonatal outcomes among all recorded singleton births in Sweden 1995–2010, with documentation of the date of last menstrual period, US-based estimated date of delivery, maternal weight, and height according to the discrepancy between pregnancy dating methods. Negative discrepancy indicates that the EDD by LMP was at an earlier date than was the EDD by US. Positive discrepancy indicates that the EDD by LMP was at a later date. A large negative discrepancy was defined as below the 10^th^ percentile, and a large positive discrepancy as above the 90^th^ percentile in the discrepancy distribution. The reference category (n = 517 657) was a discrepancy within 2 days of the median. The intermediate groups were defined as small negative and small positive discrepancies.

*Excluding deliveries by cesarean section; **Excluding elective cesarean section.

OR, odds ratio; CI, confidence interval; EDD, estimated delivery date; LMP, last menstrual period; US, ultrasound; SD, standard deviation.

### Delivery outcomes

A *negative discrepancy* between dating methods was associated with lower odds than expected for all adverse delivery outcomes related to large infants, except for shoulder dystocia. Except for emergency cesarean section, these results remained significant after adjustments in all four models (Supplementary Table 2). Correspondingly, a *positive discrepancy* was associated with higher odds than expected for most adverse delivery outcomes that were expected to be more frequent among large infants at birth (Table 1). The effect estimates for cesarean section were slightly lower in models 2 and 3 when there was a large positive discrepancy. The positive effect estimates for shoulder dystocia were not significant after adjustments (Supplementary Table 2). The results changed marginally after exclusion of SGA and LGA births (data not shown).

### Neonatal outcomes

The highest effect estimates were found for intrauterine fetal death, SGA at birth, and neonatal death in cases of a large negative discrepancy. A *negative discrepancy* between the dating methods was associated with higher odds for low Apgar score (limited to large negative discrepancy), birth asphyxia (limited to large negative discrepancy), intrauterine fetal death, neonatal death, SGA, and LGA (Table 1). The largest effect estimate was found for neonatal death: OR 2.19 (95% CI 1.91–2.50). The results remained generally significant after adjustments, except for some of the results related to LGA (Supplementary Table 2). In cases of *positive discrepancy*, the odds were lower for intrauterine fetal death (limited to small positive discrepancy) and SGA. Some of the results were marginally changed after adjustments (Supplementary Table 2). After exclusion of SGA and LGA births, the crude OR for neonatal death in cases of negative discrepancy was 1.53 (95% CI 1.28–1.84), and the OR for neonatal death was 1.18 (95% CI 1.03–1.35) (data not shown).

In the large negative discrepancy category, the rate of SGA at birth was 52% higher than in the reference category. NNT was 96, i.e. in order to find one infant with SGA, additionally 95 non-SGA pregnancies with large negative discrepancies would have to be followed up. The rate of intrauterine fetal death was 47% higher (NNT was 833), and the rate of neonatal death was 117% higher (NNT was 701) (Table 2).

**Table 2:** Number of events, risk difference, and numbers needed to treat (NNT) for adverse obstetric and neonatal outcomes according to discrepancy between pregnancy dating methods.

	Reference (n = 517657)		Large negative discrepancy (n = 119 275)				Large positive discrepancy (n = 113 236)			
	Number of events	Event rate n/100 000	Number of events	Event rate n/100 000	Risk difference	NNT	Number of events	Event rate n/100 000	Risk difference	NNT
**Pregnancy or delivery outcomes**										
Preeclampsia	16752	3236	4706	3946	0,0071	141	3564	3147	−0,0009	—
Diabetes mellitus in pregnancy	7091	1370	2374	1990	0,0062	161	1332	1176	−0,0019	—
Prolonged second stage of labor	3781	730	788	661	−0,0007	—	918	811	0,0008	1245
Delivery by forceps or vacuum extractor*	34412	6648	6996	5865	−0,0078	—	8324	7351	0,0070	142
Delivery by cesarean section	70122	13546	15019	12592	−0,0095	—	15370	13573	0,0003	3651
Delivery by emergency cesarean section**	35421	6843	7784	6526	−0,0032	—	7901	6977	0,0013	741
Shoulder dystocia*	1021	197	263	220	0,0002	4298	260	230	0,0003	3089
Postpartum hemorrhage	29413	5682	6274	5260	−0,0042	—	7226	6381	0,0070	143
Perineal laceration: third or fourth degree*	16340	3157	3220	2700	−0,0046	—	4014	3545	0,0039	258
**Neonatal outcomes**										
Apgar score < 7 at 5 minutes	2816	544	825	692	0,0015	677	537	474	−0,0007	
Birth asphyxia	4941	954	1340	1123	0,0017	592	1154	1019	0,0006	1548
Intrauterine fetal death	1314	254	446	374	0,0012	833	255	225	−0,0003	
Neonatal death (<28 days)	624	121	314	263	0,0014	701	136	120	0,0000	—
Small for gestational age (<2 SDs)	10347	1999	3632	3045	0,0105	96	1873	1654	−0,0034	
Large for gestational age (>2 SDs)	18320	3539	4619	3873	0,0033	300	4087	3609	0,0007	1423

Number of events, risk difference, and numbers needed to treat (NNT = 1/risk difference) for adverse obstetric and neonatal outcomes among all recorded singleton births in Sweden 1995–2010, with documentation of the date of last menstrual period, US-based estimated date of delivery, maternal weight, and height according to the discrepancy between pregnancy dating methods. Negative discrepancy indicates that the EDD by LMP was at an earlier date than was the EDD by US. Positive discrepancy indicates that the EDD by LMP was at a later date. A large negative discrepancy was defined as below the 10^th^ percentile, and a large positive discrepancy as above the 90^th^ percentile in the discrepancy distribution. The reference category (n = 517 657) was a discrepancy within 2 days of the median. The intermediate groups are not included in this comparison. NNT should in this context be interpreted as number needed to follow up more closely to possibly detect an adverse outcome. When NNT <1, the adverse outcome is expected to be less prevalent than in the reference category, and these numbers were omitted from the table.

*Excluding deliveries by cesarean section; **Excluding elective cesarean section.

## Discussion

### Main findings

In this large population-based cohort study, a discrepancy between EDD by LMP and EDD by US was associated with several adverse pregnancy, delivery, and neonatal outcomes. Most importantly, a large negative discrepancy was associated with higher odds for neonatal and intrauterine fetal death, as well as SGA. A positive discrepancy was associated with adverse delivery outcomes related to large infant size.

### Pregnancy outcomes

A reported association between a negative discrepancy and subsequent preeclampsia was confirmed in this population-based study^14^. In women with preeclampsia, the reason for a negative discrepancy may be early growth restriction^20^. In women with diabetes, the association with negative discrepancy may reflect longer menstrual cycles, because women with an irregular menstrual cycle have an increased risk of developing diabetes mellitus^25^. Another plausible explanation is restricted intrauterine growth in the first half of diabetic pregnancies along with catch-up growth in late pregnancy, as reported for some women with type 1 diabetes^19^. We note that diabetes mellitus, which was assessed as an outcome if registered during the studied pregnancies, may also have been present before the studied pregnancies.

### Delivery outcomes

The odds for adverse delivery outcomes varied according to the magnitude and direction of discrepancy between methods. This observation suggests that a discrepancy between methods sometimes reflects deviating fetal growth instead of imprecision in the LMP-based estimate^26–28^. Adjusting for SGA or LGA, or excluding these covariates from the analyses, changed the effect estimates only marginally. In contrast to an earlier study^29^, a positive discrepancy was not associated with an increased risk for cesarean section.

### Neonatal outcomes

A higher risk for adverse neonatal outcomes observed for pregnancies with a negative discrepancy has been described previously in part of the same study population; from a shorter time-period and with fewer neonatal outcomes evaluated^9^. In the current study, a negative discrepancy was also associated with birth asphyxia and SGA. Associations between discrepancy and the outcomes SGA and LGA will always be biased as they are defined by the US method, using fetal size as a proxy for age. Therefore, the GA will be underestimated if there is slow early fetal growth, and, as a consequence, suboptimal fetal growth later in pregnancy may be underestimated or not detected. Another consequence of underestimated GA is that labor will not be induced within the optimal pregnancy duration, as indicated by other studies^4,9^.

In this study, adjusting for SGA or LGA in the analyses had only a minor effect on the increased odds for intrauterine fetal death and neonatal death, although US-based underestimation of SGA may have diluted this effect^3^. However, excluding SGA and LGA reduced the effect estimates for intrauterine or neonatal death in cases of large negative discrepancy. This result suggests that continued decelerating or accelerating of fetal growth may contribute to the association of a discrepancy between methods with these neonatal outcomes. Negative discrepancy alone was associated with SGA, but also to a smaller extent, with LGA. The latter may indicate incorrect recording of the LMP or catch-up growth after initially slower fetal growth, which may occur in diabetic pregnancies^19^.

### Strengths and limitations

The strengths of this study are the large population-based study population and the use of information from national registers, with almost complete coverage and with prospectively collected information of high validity. The results are consistent with previous studies, but also add new knowledge because more outcomes were assessed. We also used four separate models for additional adjustments to control for possible confounding variables. One limitation was the lack of valid information regarding the regularity of menstrual cycles and when the US examinations were performed. Another possible limitation is that fetuses with congenital malformations affecting early fetal size were not excluded from the analyses, although we note that malformations occurred in only 3% of all births during the study period^30^. Malformations that resulted in termination of pregnancy or fetal death before viability were not included in the study because these were not recorded on an individual level in the national health registers. The prevalence of adverse outcomes may have been underestimated because some events or diagnoses might not have been recorded; however, assuming no association with the discrepancy categories, this should only have diluted the observed associations^16,17^.

### Implications for clinical practice and future directions

Our findings that discrepancy between the two pregnancy dating methods is associated with adverse perinatal outcomes may be useful in clinical practice for identifying pregnancies at risk of adverse outcomes. Although any discrepancy between methods may reflect an erroneous EDD estimated by LMP; it could also be a risk indicator for adverse outcomes. As a large negative discrepancy was the strongest risk indicator for intrauterine and neonatal death, and was also strongly associated with SGA at birth, pregnancies with large discrepancies may benefit the most from additional and close follow-up to lower the risk of perinatal mortality. Identifying discrepancies between dating methods may be a cost-effective way to select pregnancies that would benefit from closer monitoring^3,4,31–33^. Adding serum markers, such as PAPP-A and free β-hCG, may improve the prognostic value of identification of a discrepancy between methods, but would also add to the cost, as would implementing routine third-trimester US screening for fetal growth restriction^34–36^.

The number of pregnancies with large discrepancy between dating methods are expected to be smaller if pregnancy dating had been based predominately on first-trimester instead of second-trimester ultrasound examinations, as the variability in growth is less pronounced in early pregnancy^1,37,38^.However, no comparison between first trimester (based on crown-rump-length or biparietal diameter) and second trimester pregnancy dating could be performed in this study, as the vast majority of pregnancies included were dated at a second trimester ultrasound examination and in accordance with clinical routines during the study period. Also, the time-points of pregnancy dating by US were not recorded in the national registers during the timespan of the study. Information on date and fetal measurements are now included in national registers and will be possible to retrieve for future studies with a similar study design as this one, when data is available for large enough birth cohorts.

## Conclusions

Discrepancy between EDD by US and EDD by LMP was associated with adverse outcomes during pregnancy, delivery, and in the neonatal period. These results support the hypothesis that a smaller or larger than expected fetal size based on the date of the LMP may in some cases reflect decelerated or accelerated early fetal growth, which could later lead to size-related adverse perinatal outcomes. Even though pregnancy dating by US is generally more accurate than that by LMP, discrepancy between methods – and especially large negative discrepancy – should be noted because it may be associated with increased risks of adverse perinatal outcomes.

## Electronic supplementary material

Supplementary information
