# Supplementary material for: Discrepancy between pregnancy dating methods affects obstetric and neonatal outcomes: a population-based register cohort study
Source: Sci Rep. 2018 May 2;8:6936. doi: 10.1038/s41598-018-24894-y (PMC5932022; doi:10.1038/s41598-018-24894-y)
Supplement: Supplementary file 1 — Supplementary information [file 41598_2018_24894_MOESM1_ESM.pdf]

Title: **Discrepancy between pregnancy dating methods affects obstetric and neonatal outcomes: a population-based register cohort study**

Short title: Dating discrepancy

KEYWORDS: pregnancy dating, pregnancy, gestational age, fetal ultrasonography, prenatal, menstruation, female

Authors:

Merit Kullinger*, MD, Center for Clinical Research, Västmanland County Hospital, Västerås, Sweden.
Michaela Granfors, MD, PhD, Department of Medicine, Clinical Epidemiology Unit, Karolinska Institutet; Department of Clinical Science, Karolinska Institutet Danderyd Hospital, Stockholm, Sweden.
Helle Kieler, MD, PhD, Professor, Centre for Pharmacoepidemiology (CPE), Department of Medicine, Solna, Karolinska Institutet, Stockholm, Sweden.
Alkistis Skalkidou, MD, PhD, Professor, Department of Women's and Children's Health, Uppsala University, Sweden.

***Corresponding author:**

Merit Kullinger
Kvinnokliniken, Västmanlands sjukhus, 721 89 Västerås, Sweden
+46-21-173000, +46-73-3106360
E-mail: merit.kullinger@kbh.uu.se

Supplementary table 1. ICD-9 and ICD-10 codes for the included pregnancy, delivery, and neonatal outcomes.

**Preeclampsia (6424, 642, O14)**		
ICD-9:	6424	Mild or unspecified pre-eclampsia
	6425	Severe pre-eclampsia
ICD-10:	O14.0	Moderate pre-eclampsia
	O14.1	Severe pre-eclampsia
	O14.2	HELLP syndrome
	O14.0	Pre-eclampsia, unspecified
**Diabetes mellitus in pregnancy (6480, O24)**		
ICD-9:	6480	Diabetes mellitus complicating pregnancy, childbirth or the puerperium
ICD-10:	O24.0	Pre-existing diabetes mellitus, insulin-dependent
	O24.1	Pre-existing diabetes mellitus, non-insulin-dependent
	O24.2	Pre-existing malnutrition-related diabetes mellitus
	O24.3	Pre-existing diabetes mellitus, unspecified
	O24.4	Diabetes mellitus arising in pregnancy, including gestational diabetes mellitus NOS
	O24.9	Diabetes mellitus in pregnancy, unspecified
**Prolonged second stage of labor (6622, O63.1)**		
ICD-9:	6622	Prolonged second stage of labor
ICD-10:	O63.1	Prolonged second stage of labor
**Delivery by forceps or vacuum extractor (6695, O81)**		
ICD-9:	6695	Forceps or vacuum extractor delivery
ICD-10:	O81.0	Low forceps delivery
	O81.1	Mid-cavity forceps delivery
	O81.2	Mid-cavity forceps with rotation
	O81.3	Other and unspecified forceps delivery
	O81.4	Vacuum extractor delivery
	O81.5	Delivery by combination of forceps and vacuum extractor
**Delivery by cesarean section (6697, O82)**		
ICD-9:	6697	Cesarean delivery
ICD-10:	O82.0	Delivery by elective caesarean section
	O82.1	Delivery by emergency caesarean section
	O82.2	Delivery by caesarean hysterectomy
	O82.8	Other single delivery by caesarean section
	O82.9	Delivery by caesarean section, unspecified
**Shoulder dystocia (6604, O66.0)**		
ICD-9:	6604	Shoulder dystocia during labor and delivery
ICD-10:	O66.0	Obstructed labor due to shoulder dystocia, impacted shoulders
**Postpartum hemorrhage (666, O72)**		
ICD-9:	6660	Third-stage postpartum hemorrhage
	6661	Other immediate postpartum hemorrhage
	6662	Delayed and secondary postpartum hemorrhage
	6663	Postpartum coagulation defects
ICD-10:	O72.0	Third-stage hemorrhage associated with retained, trapped or adherent placenta
	O72.1	Other immediate postpartum hemorrhage, including atonic hemorrhage
	O72.2	Delayed and secondary postpartum hemorrhage
	O72.3	Postpartum coagulation defects

Perineal laceration: third or fourth degree (6642, 6643, O70.2, O70.3)

ICD-9:	6642	Third-degree perineal laceration during delivery
	6643	Fourth-degree perineal laceration during delivery
ICD-10:	O70.2	Third degree perineal laceration during delivery
	O70.3	Fourth degree perineal laceration during delivery

Birth asphyxia (7685, P21.0)

ICD-9:	7685	Severe birth asphyxia
ICD-10:	P21.0	Severe birth asphyxia

Supplementary table 2. Risks of specific adverse obstetric outcomes and neonatal outcomes according to the size of the discrepancy between dating by ultrasound examination and by last menstrual period (n = 1 100 049): crude and four adjusted models.

	**Prevalence** (n/100 000 births)	**Large negative discrepancy** n = 119 275		**Small negative discrepancy** n = 238 929		**Small positive discrepancy** n = 110 952		**Large positive discrepancy** n = 113 236	
		OR	(95% CI)	OR	(95% CI)	OR	(95% CI)	OR	(95% CI)
**Pregnancy or delivery outcomes**									
Preeclampsia									
Unadjusted model	3377	1.23	(1.19–1.27)	1.13	(1.10–1.16)	0.96	(0.93–1.00)	0.97	(0.94–1.01)
Model 1		1.19	(1.15–1.23)	1.09	(1.06–1.12)	0.98	(0.94–1.02)	0.98	(0.95–1.02)
Model 2		1.20	(1.15–1.24)	1.10	(1.07–1.13)	0.98	(0.94–1.02)	0.98	(0.94–1.02)
Model 3		1.18	(1.14–1.22)	1.09	(1.06–1.12)	0.98	(0.94–1.02)	0.98	(0.94–1.02)
Model 4		1.13	(1.09–1.17)	1.06	(1.03–1.09)	1.00	(0.96–1.04)	1.00	(0.96–1.04)
Diabetes mellitus in pregnancy									
Unadjusted model	1459	1.46	(1.39–1.53)	1.20	(1.16–1.25)	0.87	(0.82–0.93)	0.86	(0.81–0.91)
Model 1		1.43	(1.36–1.51)	1.20	(1.15–1.25)	0.88	(0.83–0.93)	0.85	(0.79–0.90)
Model 2		1.44	(1.36–1.51)	1.21	(1.16–1.26)	0.87	(0.82–0.93)	0.84	(0.79–0.89)
Model 3		1.42	(1.35–1.49)	1.20	(1.15–1.25)	0.88	(0.82–0.93)	0.84	(0.79–0.89)
Model 4		1.41	(1.34–1.48)	1.20	(1.15–1.25)	0.87	(0.82–0.93)	0.84	(0.78–0.89)
Prolonged second stage of labor									
Unadjusted model	720	0.90	(0.84–0.98)	0.88	(0.83–0.94)	1.10	(1.02–1.18)	1.11	(1.03–1.19)
Model 1		0.92	(0.84–0.99)	0.88	(0.83–0.94)	1.10	(1.02–1.18)	1.11	(1.03–1.20)
Model 2		0.92	(0.84–0.99)	0.88	(0.83–0.94)	1.09	(1.01–1.18)	1.11	(1.03–1.20)
Model 3		0.92	(0.84–0.99)	0.88	(0.83–0.94)	1.10	(1.01–1.18)	1.11	(1.03–1.20)
Model 4		0.92	(0.85–1.00)	0.88	(0.83–0.94)	1.09	(1.01–1.18)	1.10	(1.02–1.19)
Delivery by forceps or vacuum extractor*									
Unadjusted model	6564	0.88	(0.85–0.90)	0.90	(0.88–0.92)	1.10	(1.07–1.13)	1.11	(1.09–1.14)
Model 1		0.89	(0.87–0.92)	0.91	(0.89–0.93)	1.10	(1.07–1.13)	1.13	(1.10–1.16)
Model 2		0.91	(0.88–0.93)	0.92	(0.90–0.94)	1.08	(1.05–1.10)	1.09	(1.06–1.12)
Model 3		0.91	(0.88–0.93)	0.92	(0.90–0.94)	1.08	(1.05–1.10)	1.09	(1.06–1.12)
Model 4		0.91	(0.88–0.93)	0.92	(0.90–0.94)	1.08	(1.05–1.10)	1.09	(1.07–1.12)
Delivery by cesarean section									
Unadjusted model	13 289	0.92	(0.90–0.94)	0.93	(0.92–0.95)	1.02	(1.00–1.03)	1.00	(0.98–1.02)
Model 1		0.96	(0.94–0.98)	0.95	(0.94–0.97)	1.00	(0.98–1.02)	0.98	(0.96–1.00)
Model 2		0.97	(0.95–0.99)	0.96	(0.95–0.97)	0.99	(0.97–1.01)	0.97	(0.95–0.99)
Model 3		0.95	(0.93–0.97)	0.95	(0.94–0.97)	1.00	(0.98–1.02)	0.97	(0.95–0.99)
Model 4		0.93	(0.91–0.95)	0.94	(0.93–0.96)	1.00	(0.98–1.02)	0.98	(0.96–1.00)
Delivery by emergency cesarean section**									
Unadjusted model	6778	0.95	(0.93–0.97)	0.95	(0.94–0.97)	1.03	(1.00–1.06)	1.02	(1.00–1.05)
Model 1		0.97	(0.94–1.00)	0.97	(0.95–0.99)	1.02	(1.00–1.05)	1.02	(0.99–1.04)
Model 2		0.98	(0.96–1.01)	0.98	(0.96–1.00)	1.01	(0.98–1.03)	0.99	(0.97–1.02)
Model 3		0.97	(0.94–1.00)	0.97	(0.95–0.99)	1.01	(0.98–1.04)	1.00	(0.97–1.02)
Model 4		0.96	(0.93–0.98)	0.97	(0.95–0.99)	1.01	(0.99–1.04)	1.00	(0.97–1.02)
Shoulder dystocia*									
Unadjusted model	202	1.12	(0.97–1.28)	0.95	(0.85–1.07)	1.02	(0.88–1.18)	1.16	(1.01–1.33)
Model 1		1.12	(0.97–1.29)	0.98	(0.87–1.10)	1.07	(0.92–1.24)	1.13	(0.97–1.30)
Model 2		1.16	(1.00–1.34)	1.01	(0.90–1.14)	1.02	(0.88–1.19)	1.06	(0.91–1.22)
Model 3		1.13	(0.98–1.31)	1.00	(0.89–1.13)	1.03	(0.88–1.20)	1.07	(0.92–1.23)
Model 4		1.13	(0.97–1.31)	1.00	(0.89–1.13)	1.03	(0.88–1.19)	1.05	(0.90–1.22)
Postpartum hemorrhage									
Unadjusted model	5661	0.92	(0.90–0.95)	0.93	(0.91–0.95)	1.06	(1.04–1.09)	1.13	(1.10–1.16)
Model 1		0.93	(0.90–0.95)	0.93	(0.91–0.95)	1.06	(1.03–1.09)	1.13	(1.10–1.16)
Model 2		0.92	(0.89–0.95)	0.92	(0.90–0.94)	1.08	(1.05–1.11)	1.15	(1.12–1.18)
Model 3		0.92	(0.89–0.94)	0.92	(0.90–0.94)	1.08	(1.05–1.11)	1.15	(1.12–1.18)
Model 4		0.92	(0.89–0.95)	0.92	(0.90–0.94)	1.07	(1.04–1.11)	1.15	(1.12–1.18)
Perineal laceration: third or fourth degree*									
Unadjusted model	3100	0.85	(0.82–0.88)	0.88	(0.85–0.90)	1.12	(1.08–1.16)	1.13	(1.09–1.17)
Model 1		0.87	(0.83–0.91)	0.88	(0.85–0.91)	1.13	(1.09–1.17)	1.15	(1.11–1.19)
Model 2		0.88	(0.85–0.92)	0.89	(0.86–0.92)	1.11	(1.07–1.15)	1.12	(1.08–1.16)
Model 3		0.88	(0.85–0.92)	0.89	(0.86–0.92)	1.11	(1.07–1.15)	1.12	(1.08–1.16)
Model 4		0.89	(0.85–0.92)	0.89	(0.87–0.92)	1.10	(1.06–1.15)	1.12	(1.08–1.16)
**Neonatal outcomes**									
Apgar score <7 at 5 minutes									
Unadjusted model	557	1.18	(1.09–1.27)	1.03	(0.97–1.10)	0.91	(0.83–1.00)	1.00	(0.91–1.10)
Model 1		1.18	(1.09–1.29)	1.03	(0.96–1.10)	0.90	(0.81–0.99)	0.96	(0.87–1.06)
Model 2		1.21	(1.12–1.32)	1.06	(0.99–1.13)	0.88	(0.79–0.97)	0.93	(0.84–1.03)
Model 3		1.20	(1.10–1.30)	1.05	(0.98–1.12)	0.88	(0.79–0.97)	0.93	(0.84–1.03)
Model 4		1.17	(1.08–1.27)	1.04	(0.97–1.12)	0.88	(0.80–0.98)	0.94	(0.85–1.04)
Birth asphyxia									
Unadjusted model	987	1.18	(1.11–1.25)	1.03	(0.98–1.08)	1.02	(0.96–1.09)	1.07	(1.00–1.14)
Model 1		1.18	(1.10–1.25)	1.00	(0.95–1.06)	1.01	(0.94–1.09)	1.05	(0.97–1.12)
Model 2		1.20	(1.13–1.28)	1.03	(0.97–1.08)	0.99	(0.92–1.06)	1.01	(0.94–1.08)
Model 3		1.19	(1.12–1.27)	1.02	(0.97–1.08)	0.99	(0.92–1.06)	1.01	(0.94–1.08)
Model 4		1.17	(1.10–1.25)	1.01	(0.95–1.06)	0.99	(0.93–1.07)	1.01	(0.94–1.09)
Intrauterine fetal death									
Unadjusted model	274	1.47	(1.32–1.64)	1.24	(1.14–1.36)	0.87	(0.75–0.99)	0.89	(0.77–1.01)
Model 1		1.49	(1.33–1.67)	1.21	(1.10–1.34)	0.86	(0.74–1.00)	0.87	(0.75–1.00)
Model 2		1.49	(1.33–1.67)	1.22	(1.10–1.34)	0.87	(0.75–1.00)	0.87	(0.75–1.01)
Model 3		1.48	(1.32–1.66)	1.21	(1.10–1.34)	0.87	(0.75–1.00)	0.87	(0.75–1.01)
Model 4		1.35	(1.20–1.52)	1.15	(1.04–1.27)	0.92	(0.79–1.06)	0.91	(0.79–1.06)
Neonatal death									
Unadjusted model	141	2.19	(1.91–2.50)	1.26	(1.10–1.43)	0.89	(0.73–1.08)	1.00	(0.82–1.20)
Model 1		2.22	(1.91–2.56)	1.24	(1.08–1.42)	0.81	(0.64–1.00)	0.91	(0.74–1.12)
Model 2		2.27	(1.96–2.63)	1.27	(1.10–1.46)	0.78	(0.63–0.97)	0.88	(0.71–1.07)
Model 3		2.24	(1.94–2.60)	1.26	(1.10–1.45)	0.78	(0.63–0.97)	0.88	(0.71–1.07)
Model 4		2.03	(1.74–2.37)	1.13	(0.97–1.31)	0.80	(0.63–1.00)	0.90	(0.73–1.11)
Small for gestational age									
Unadjusted model	2169	1.54	(1.48–1.60)	1.31	(1.26–1.35)	0.81	(0.77–0.86)	0.82	(0.78–0.87)
Model 1		1.50	(1.44–1.56)	1.29	(1.25–1.34)	0.82	(0.77–0.86)	0.80	(0.76–0.85)
Model 2		1.51	(1.45–1.57)	1.30	(1.26–1.35)	0.81	(0.77–0.86)	0.80	(0.76–0.84)
Model 3		1.48	(1.42–1.54)	1.29	(1.25–1.34)	0.81	(0.77–0.86)	0.80	(0.76–0.84)
Model 4		1.49	(1.42–1.55)	1.29	(1.25–1.34)	0.82	(0.77–0.86)	0.80	(0.76–0.84)

Large for gestational age									
Unadjusted model	3617	1.10	(1.06–1.14)	1.04	(1.01–1.07)	1.02	(0.99–1.06)	1.02	(0.99–1.06)
Model 1		1.07	(1.03–1.11)	1.03	(1.00–1.06)	1.03	(0.99–1.07)	1.03	(0.99–1.07)
Model 2		1.07	(1.03–1.11)	1.03	(1.00–1.06)	1.03	(1.00–1.07)	1.04	(1.00–1.08)
Model 3		1.04	(1.00–1.08)	1.01	(0.99–1.04)	1.04	(1.00–1.08)	1.05	(1.01–1.09)
Model 4		1.05	(1.01–1.09)	1.02	(0.99–1.05)	1.04	(1.00–1.08)	1.04	(1.01–1.08)

Logistic regression-derived crude ORs and 95% CIs for adverse obstetric and neonatal outcomes among all recorded singleton births in Sweden 1995–2010 according to the discrepancy between pregnancy dating methods. Information was obtained from the Medical Birth Register, and only those with a record of the date of last menstrual period (LMP), ultrasound (US)-based estimated date of delivery (EDD), maternal weight, and height were included in the analyses. A negative discrepancy indicates that EDD by LMP was at an earlier date than was the EDD by US; a positive discrepancy indicates that the EDD by LMP was at a later date than was the EDD by US. A large negative discrepancy was defined as below the 10th percentile, and a large positive discrepancy as above the 90th percentile in the discrepancy distribution. The reference category (n = 517 657) was defined as discrepancies within 2 days of the median. The intermediate groups were defined as small negative and small positive discrepancies.

Effect estimates are derived from the crude and four adjusted models as follows:
Model 1: adjusted for BMI (weight (kg)/height (m^2) <18.5 kg/m^2 or >30 kg/m^2, maternal age <20 years or >30 years, smoking or snuff use, living without a partner, or not being employed.
Model 2: same as model 1, but adding fetal sex, with female as the reference category.
Model 3: same as model 2, but adding the diagnosis of diabetes mellitus and preeclampsia during the current pregnancy as separate covariates.
Model 4: same as model 3, but adding SGA or LGA at birth.

*excluding deliveries by cesarean section; **excluding elective cesarean section.

OR, odds ratio; CI, confidence interval
